# Microneutralization Assay Titres Correlate with Protection against Seasonal Influenza H1N1 and H3N2 in Children

**DOI:** 10.1371/journal.pone.0131531

**Published:** 2015-06-24

**Authors:** Chris P. Verschoor, Pardeep Singh, Margaret L. Russell, Dawn M. E. Bowdish, Angela Brewer, Louis Cyr, Brian J. Ward, Mark Loeb

**Affiliations:** 1 McMaster Immunology Research Centre, McMaster University, Hamilton, Ontario, Canada; 2 Department of Pathology and Molecular Medicine, McMaster University, Hamilton, Ontario, Canada; 3 Michael G DeGroote Institute for Infectious Disease Research, McMaster University, Hamilton, Ontario, Canada; 4 Department of Clinical Epidemiology and Biostatistics, McMaster University, Hamilton, Ontario, Canada; 5 Cumming School of Medicine, University of Calgary, Calgary, Alberta, Canada; 6 Research Institute of the McGill University Health Center, Montreal, QC, Canada; University of Hong Kong, HONG KONG

## Abstract

Although the microneutralization (MN) assay has been shown to be more sensitive than the hemagglutination inhibition (HAI) assay for the measurement of humoral immunity against influenza viruses, further evidence relating MN titres to protective efficacy against infection is needed. Serum antibodies against seasonal H1N1 and H3N2 influenza were measured in children and adolescents (n = 656) by MN and hemagglutination inhibition (HAI) assays. Compared to HAI, the MN assay is more sensitive in detecting serum antibodies and estimates of protective effectiveness against PCR-confirmed infection were higher for both subtypes. Given our findings, the MN assay warrants further consideration as a formal tool for the routine evaluation of vaccine-induced antibody responses.

## Introduction

The quantification of serum antibodies to microbial antigens, particularly following immunization, has long been used to assess the likelihood of protection against infection. One of the classical ways of measuring such antibodies has been the hemagglutination inhibition (HAI) assay, which assesses the ability of test sera to prevent the agglutination of red blood cells by particulate antigens (eg. virions). With regard to vaccine-induced protection against influenza infection, it is widely thought that an HAI titre ≥1:40 corresponds to a 50% reduction in the prevalence of infection [1]. However, as previously discussed [2], the evidence for this cut-off value is derived largely from adult cohorts, and may not apply to children, adolescents or the elderly. For example, Black and colleagues (2011) estimated that a more appropriate HAI cut-off for 50% protection in children would instead be 1:110 [2]. Others have reported that 1:40 is likely too low of an HAI titre cut-off for adequate protection in the elderly as well [3].

The HAI assay has also been criticised for its overall insensitivity, thereby underestimating seroprevalence in a given population. For example, a recent study in England reported that baseline (pre-vaccination) HAI titres for pandemic influenza H1N1 were below the limit of detection (<1:8) in 83% of individuals 10–50 years old, and in 62% of individuals 50–80 years old [4]. The inability to define baseline levels in such a large proportion of individuals hinders not only the evaluation of baseline protection, but also the ability to accurately estimate seroconversion rates following vaccination.

Given the limitations of HAI, the microneutralization (MN) assay is an attractive alternative for the assessment of baseline serostatus as well as the humoral response following vaccination or natural infection. This assay is based on the ability of serum antibodies to prevent infection of mammalian cells *in vitro*, and as such, represents a more mechanistically relevant estimation of antibody-mediated protection compared to HAI. Just as important, results from the MN assay are usually highly correlated with HAI titres, but of considerably higher sensitivity; for example, previous estimates indicate that an HAI titre of 1:40 corresponds to an MN titre of approximately 1:160 [1,5,6]. Despite a general consensus that the MN assay is likely to be a superior tool for the evaluation of vaccine-induced responses [1,7], data describing the relationship between MN titres and protection against influenza infection are sparse. The preference for HAI data is largely explained by the greater technical complexity and cost of the MN assay, the requirement for live virus and difficulties in standardization across sites. These issues have limited the use of the MN assay as a formal tool in the estimation of protection against influenza [8].

In the present study, we used sera collected from a prospective cohort of 656 children and adolescents 3–15 years of age to measure HAI and MN antibody titres against influenza H1N1 and H3N2. These data were then used to estimate cut-off titres predictive of protective effectiveness against infection during the ensuing influenza season.

## Materials and Methods

### Participants

A total of 656 healthy Hutterite children and adolescents 3–15 years of age from Manitoba and Alberta enrolled in a randomized controlled trial evaluating the effect of influenza vaccination on infection prevalence (clinicaltrials.gov: NCT00877396; isrctn.org: ISRCTN15363571) were included in this study. This work was approved by the McMaster Research Ethics Review Board and written informed consent was obtained for all participants and/or their legal guardians. The general study design has been previously described [9]. Briefly, participants were randomly assigned by Hutterite colony (n = 42) to receive either the inactivated seasonal trivalent influenza vaccine (TIV; n = 309; Vaxigrip, Sanofi Pasteur, Lyon, France) or the hepatitis A vaccine (HAV; n = 347; Avaxim-Pediatric, Sanofi Pasteur), and blood specimens were drawn at least 3–5 weeks post-vaccination. Individuals in colonies randomized to the TIV group received a 0.5-mL dose of the study vaccine intramuscularly. Those younger than 9 years who were previously unvaccinated at the time of immunization received a second 0.5-mL dose of the TIV 4 weeks after the first vaccine. In colonies receiving the HAV, individuals were immunized in a manner that mimicked the influenza immunization schedule to maintain blinding, only those younger than 9 years of age who were previously unvaccinated for influenza received a second 0.5-mL injection of sterile saline. The TIV used in Canada that year contained antigens from A/Brisbane/59/2007 (H1N1)-like, A/Brisbane/10/2007 (H3N2)-like and B/Florida/4/2006-like viruses; both A/Brisbane/59/2007 (H1N1)-like and A/Brisbane/10/2007 (H3N2)-like have been previously shown to significantly match circulating strains during the 2009 North American influenza season [10]. Vaccine administration start dates ranged from October 30, 2008, for colonies in Alberta to November 13, 2008, for colonies in Manitoba. Infection monitoring was conducted twice weekly during the influenza season (December 28, 2008, to June 23, 2009) and positive cases were confirmed by PCR of nasal swabs, as previously described [9].

### Antibody tests

Hemagglutination inhibition titres were determined by standard methods. Briefly, turkey erythrocytes were incubated with reference antigens for A/Brisbane/59/2007 (H1N1)-like and A/Brisbane/10/2007 (H3N2)-like viruses. The HAI assay was completed and interpreted as previously reported [9,11]. MN titres were determined as previously described [12]. Briefly, H1N1 and H3N2 Influenza virus stocks were prepared in Madin-Darby canine kidney (MDCK) cells (ATCC CCL-34). The A/Brisbane/59/2007 (H1N1)-like was supplied by Y. Li (National Microbiology Laboratory, Winnipeg, MB) while A/Brisbane/10/2007 (H3N2)-like was purchased from BEI (Manassas, VA). Sera were heat-inactivated (56°C for 30 min) and stored at −20°C until use. MDCK cells were seeded into flat-bottom 96-well plates (30,000 to 45,000/well) in HyClone SFM4MegaVir medium (Thermo Scientific, Waltham, MA) supplemented with 10 μg/ml gentamicin (Gibco Life Technologies, Burlington, ON), 0.25 μg/ml amphotericin B (Gibco Life Technologies), 100,000 U/ml penicillin G (Sigma, St. Louis, MO), and 10 μg/ml glutamine (Wisent, St. Bruno, QC) to achieve confluent cell monolayers, which were used within 3 days of confluence. Two-fold serial dilutions of serum starting at 1:10 in MegaVir were incubated with 100 50% tissue culture infective doses (TCID_50_) of virus for 2 h at 37°C with 5% CO_2_. The serum/virus was then added to MDCK cells in MegaVir medium with 1× TPCK (tolylsulfonyl phenylalanyl chloromethyl ketone)-treated trypsin. After 3 h at 37°C with 5% CO_2_, the medium in each well was refreshed with MegaVir medium with 0.75× TPCK-treated trypsin. The cells were observed for the presence of cytopathic effect (3 to 5 days), and the MN titer was defined as the highest dilution to retain a confluent cell monolayer. The assay was repeated if the sample replicates differed by more than one dilution. MN titers below the limit of detection (<1:10) were assigned a value of 1:5 for statistical analysis.

### Statistical analysis

Summary statistics for antibody titres are presented as median (25^th^ and 75^th^ quartile), and associations between log-transformed antibody titres and age and sex were determined by linear regression, adjusting for multiple testing using the Benjamini-Hochberg procedure [13]. Correlations between HAI and MN derived log-transformed titres were performed by linear regression. Protective effectiveness at each titre threshold was determined using Cox’s proportional hazards model, adjusting for clustering using a robust sandwich estimator, and is defined as (1-hazard ratio) x 100%. Hazard ratios represent the ratio of infection rates for individuals greater than or equal to a given titre threshold, and those with less than the given threshold. Significance was calculated using standard error estimates from the regression model and adjusted for multiple testing using the Benjamini-Hochberg procedure. For example, at a cut-off of 1:80, a hazard ratio was calculated for individuals with a titre greater than or equal to 1:80, as compared to those with a titre less than 1:80. All analyses were performed in R version 3.0.1.

## Results

Influenza infection was monitored in a cohort of 656 children and adolescents 3–15 years of age from December 28, 2008, to June 23, 2009 and confirmed by nasal swab PCR. For individuals receiving the inactivated seasonal trivalent influenza vaccine (TIV, n = 309), 7 (2.3%) were infected with pandemic H1N1 (pH1N1), 0 with seasonal H1, 7 (2.3%) with seasonal H3, and 13 (4.2%) with subtype B. For individuals receiving the hepatitis A vaccine (HAV, n = 347), 0 were infected with pH1N1, 16 (4.6%) with seasonal H1, 19 (5.5%) with seasonal H3, and 33 (9.5%) with subtype B.

**Fig 1:**
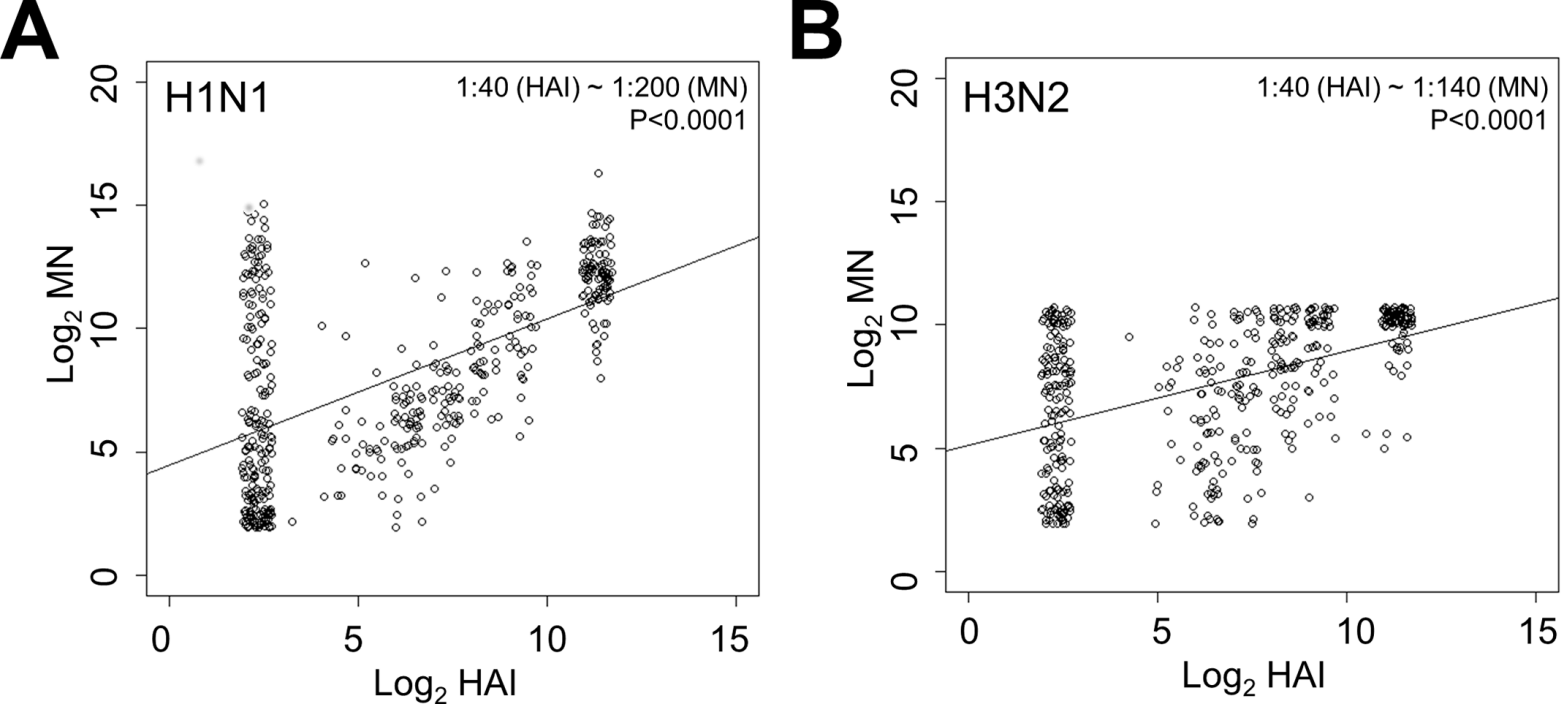
Microneutralization (MN) and hemagglutination inhibition (HAI) titres are highly correlated for seasonal influenza subtypes H1N1 and H3N2. Log-transformed MN and HAI titres for subtypes A) H1N1 (A/Brisbane/59/2007) and B) H3N2 (A/Brisbane/10/2007) are presented, along with the significance of correlation as determined by linear regression.

Serum antibody titres against seasonal influenza H1N1 (A/Brisbane/59/2007) and H3N2 (A/Brisbane/10/2007) were determined following vaccination using the HAI and MN assays. No associations between log-transformed titres with age or sex were observed after adjusting for multiple testing by the Benjamini-Hochberg procedure. Median titres (25^th^–75^th^ quartiles) in TIV and HAV vaccinated individuals for H1N1 and H3N2 antibodies were as follows: HAI, 40 (5–640) and 160 (5–640), respectively; MN, 320 (40–2560) and 320 (40–1280), respectively. A significant degree of correlation (H1N1, β = 0.588, p<0.0001; H3N2, β = 0.389, p<0.0001) was observed between titres derived from the HAI and MN assay (Fig 1A and 1B). Based on these comparisons, an HAI titre of 1:40 for H1N1 and H3N2 related to MN titres of approximately 1:200 (H1N1, Fig 1A) and 1:140 (H3N2, Fig 1B), respectively. Furthermore, only 11 and 9% of participants following vaccination had MN titres below the limit of detection (<1:10) for H1N1 and H3N2, respectively, whereas 44 and 33% of participants had undetectable HAI titres.

**Fig 2:**
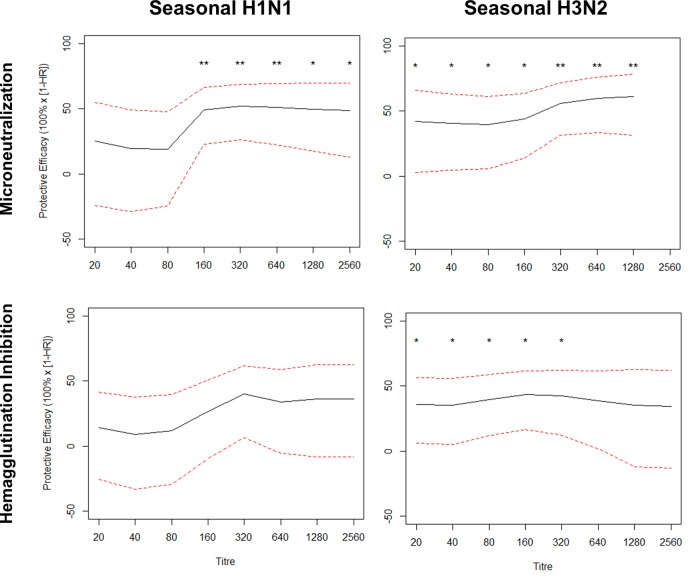
Protective effectiveness of antibody titre cut-offs against seasonal influenza H1N1 and H3N2 is greater when estimated using the microneutralization (MN) assay, as compared to the hemagglutination inhibition (HAI) assay. Protective effectiveness against PCR-confirmed influenza was compared at different MN and HAI titre cut-offs for seasonal H1N1 (A/Brisbane/59/2007) and H3N2 (A/Brisbane/10/2007). The hazard ratio represents the risk at cut-offs greater than or equal to a given titre, relative to levels less than the cut-off, and was calculated using Cox’s proportional hazards model, adjusting for donor colony using a robust sandwich estimator. Dotted lines represent the 95% confidence interval and p-values (adjusted using the Benjamini-Hochberg procedure) were calculated using standard error estimates from the regression model. **, p<0.01; *, p<0.05.

Data on estimates of the protective effectiveness of MN antibody titres against influenza infection is sparse. As such, it was our goal to compare seroprotection estimates for seasonal H1N1 and H3N2 infection based upon antibody titres measured by the MN and HAI assays. We found that protective effectiveness estimates related to MN titres were consistently higher than those for HAI titres. For H1N1 MN titres, effectiveness estimates plateaued at 50% at a titre of 1:160 (95% confidence interval (CI), 23–67; p<0.01), while for HAI titres, effectiveness plateaued at 40% at a titre of 1:320 (95% CI, 6–62; p>0.05; Fig 2A). For H3N2, the MN assay plateaued at 60% effectiveness at a titre of 1:320 (95% CI, 31–72; p<0.01). In contrast, the effectiveness curve from H3N2 HAI did not plateau but was maximal at an effectiveness of approximately 40% at a titre of 1:160 (95% CI, 17–62; p<0.05; Fig 2B).

## Discussion

Although HAI has been the most commonly used assay to measure serum antibodies against influenza, it has been criticized for its insensitivity as well as its mechanistic relevance to natural cellular infection. In this respect, the MN assay is viewed as an attractive alternative to HAI. However, little data is available regarding the correlation of MN titres to protection against influenza infection. Hence, the goal of the following study was to compare titres derived from the HAI and MN assays, and estimate their protective effectiveness against season H1N1 and H3N2 influenza infection in a cohort of children and adolescents aged 3–15.

We found that HAI and MN were significantly correlated, where an HAI titre of 1:40 for H1N1 and H3N2 related to MN titres of approximately 1:200 and 1:140, respectively. These findings are very similar to previous estimates, that an HAI titre of 1:40 corresponds to an MN titre of approximately 1:160 [1,5,6]. Furthermore, our observations also support previous findings that the MN assay is a more sensitive assay, as only 11 and 9% of participants following vaccination had MN titres below the limit of detection for H1N1 and H3N2, respectively, whereas 44 and 33% of participants had undetectable HAI titres. A major finding of the current study was that MN titres not only correlate with protection against H1N1 and H3N2 infection, but the protective efficacy estimates for the MN assay were consistently higher than HAI. Tsang and colleagues [14] have recently reported similar results regarding the ability of MN and HAI titres to predict protection against infection in a large cohort of children and adults. They found that the age-adjusted HAI and MN titres corresponding to 50% protection against seasonal H3N2 influenza was 1:260 and 1:42, respectively.

A drawback of the current study was that very few individuals (<20) were sampled after follow-up, thereby limiting our ability to adequately account for the effect of antibody waning on our estimates of protective efficacy. In our study, individuals who developed an infection and were in the vaccinated or hepatitis groups did so in a median of 97 (8–189) and 106 (17–150) days after follow-up collection, respectively. Considering the observations of Ng and colleagues [15], who found that log_2_ HAI titres decrease at a rate of 0.135 to 0.315 per month depending on the subtype considered, it is therefore possible that antibody waning did have an effect on our estimates of protective efficacy and thus, the conclusions drawn regarding the performance of the MN and HAI assays. Given that the MN assay has a greater sensitivity for detecting antibody titres against influenza, it would be of great interest in future studies to estimate the effects of antibody waning on correlation of protection estimates from both the MN and HAI assays.

In summary, we have provided much needed evidence regarding the value of the MN assay for estimating protection from influenza infection, compared to the traditional HAI assay. The MN assay is not only more sensitive in the detection of both H1N1 and H3N2 antibodies, protection effectiveness estimates against PCR-confirmed influenza infection are higher with the MN assay at similar titre thresholds. Although issues with inter-laboratory standardization remain, our observations suggest that the MN assay should be given more consideration as a formal tool for the evaluation of influenza serostatus, especially in populations expected to have relatively low baseline and/or post-vaccination antibody titres.
