# Correction: Microneutralization Assay Titres Correlate with Protection against Seasonal Influenza H1N1 and H3N2 in Children

**DOI:** 10.1371/journal.pone.0163830

**Published:** 2016-09-22

**Authors:** Chris P. Verschoor, Pardeep Singh, Margaret L. Russell, Dawn M. E. Bowdish, Angela Brewer, Louis Cyr, Brian J. Ward, Mark Loeb

There is an error in Fig 2: the HAI titres for H1N1 could not be determined and the graph was therefore revised. Please find a corrected version of Fig 2 below.

**Fig 2:**
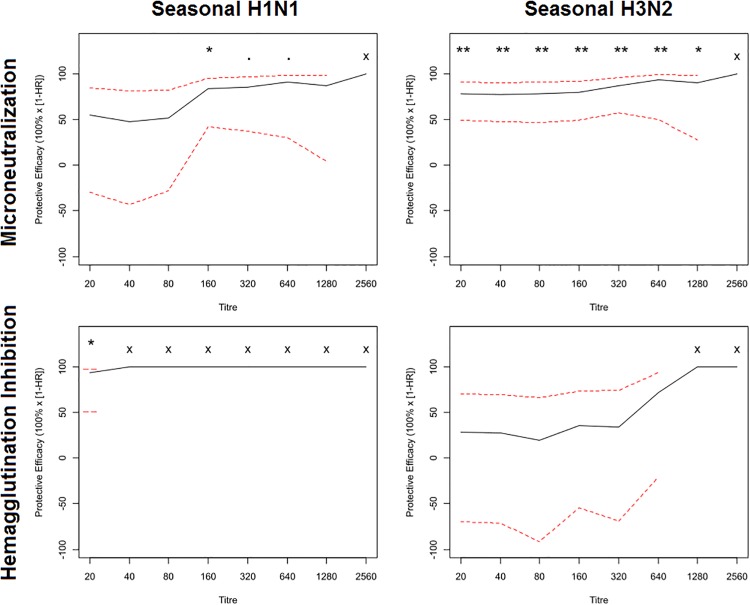
Protective effectiveness of antibody titre cut-offs against seasonal influenza H1N1 and H3N2 is greater when estimated using the microneutralization (MN) assay, as compared to the hemagglutination inhibition (HAI) assay. Protective effectiveness against PCR-confirmed influenza was compared at different MN and HAI titre cut-offs for seasonal H1N1 (A/Brisbane/59/2007) and H3N2 (A/Brisbane/10/2007). The hazard ratio represents the risk at cut-offs greater than or equal to a given titre, relative to levels less than the cut-off, and was calculated using Cox’s proportional hazards model, adjusting for donor colony using a robust sandwich estimator. Dotted lines represent the 95% confidence interval and p-values (adjusted using the Benjamini-Hochberg procedure) were calculated using standard error estimates from the regression model.

Additionally, the description of Fig 2 in the third paragraph of the Results should read as follows:

We found that protective effectiveness estimates related to MN titres were often higher than those for HAI titres. For H1N1 MN titres, effectiveness estimates plateaued at 85% at a titre of 1:160 (95% confidence interval (CI), 42–95; p<0.05), while HAI titres could not be determined because of inadequate sample size (Fig 2A). For H3N2, the MN assay plateaued at 90% effectiveness at a titre of 1:320 (95% CI, 57–96; p<0.01). In contrast, the effectiveness curve from H3N2 HAI did not plateau but was maximal approaching an effectiveness of approximately 75% at a titre of 1:640 (95% CI, -20-93; p>0.05; Fig 2B)

In keeping with the corrections of Fig 2, the third sentence of the Abstract should read:

Compared to HAI, the MN assay is more sensitive in detecting serum antibodies and estimates of protective effectiveness against PCR-confirmed infection were higher for seasonal H3N2.

The third sentence of the third paragraph of the Discussion should read:

Considering the observations of Ng and colleagues [15], who found that log_2_ HAI titres decrease at a rate of 0.135 to 0.315 per month depending on the subtype considered, it is therefore possible that antibody waning did have an effect on our estimates of H3N2 protective efficacy and thus, the conclusions drawn regarding the performance of the MN and HAI assays.

The second sentence of the fourth paragraph of the Discussion should read:

The MN assay is not only more sensitive in the detection of both H1N1 and H3N2 antibodies, protection effectiveness estimates against H3N2 PCR-confirmed influenza infection are higher with the MN assay at similar titre thresholds.
